# Intergenerational Parenting Styles and Children’s Problem Behaviors: The Mediating Role of the Grandparent–Parent Relationship

**DOI:** 10.3390/bs15081029

**Published:** 2025-07-29

**Authors:** Furong Lu, Feixia Zhang, Rong Lyu, Xinru Wu, Yuyu Wang

**Affiliations:** 1 School of Education Science, Shanxi University, Taiyuan 030006, China; lufr@sxu.edu.cn (F.L.); zhangfeixia1@sxu.edu.cn (F.Z.); lvrong@sxu.edu.cn (R.L.); wuxinru@sxu.edu.cn (X.W.); 2 Centre for Psychological Health Education, Henan University of Science and Technology, Luoyang 471023, China

**Keywords:** intergenerational parenting styles, grandchildren, problem behaviors, grandparent–parent relationship, latent profile analysis

## Abstract

In China, grandparents play a significant role in childrearing. This study aims to identify latent profiles of intergenerational parenting styles and explore their impact on grandchildren’s behavioral outcomes. A total of 1432 Chinese children (*M*_age_ = 12.58 years; 45.25% boys) completed questionnaires assessing perceived grandparenting styles, while fathers and mothers independently reported their own parenting practices. Latent Profile Analysis (LPA) identified three distinct parenting profiles: “Grandparents Positive–Parents Negative” (GP–PN, 18.37%), “Grandparents Positive–Parents Positive” (GP–PP, 59.15%), and “Grandparents Negative–Parents Positive” (GN–PP, 22.48%). Regression analyses revealed that the number of siblings, grade level, and grandparent type could significantly predict profile membership. Notably, consistent and positive intergenerational parenting styles were associated with fewer problem behaviors in children. Furthermore, the relationship between parenting profiles and problem behaviors was mediated by the quality of the grandparent–parent relationship. These findings suggested that grandparents and parents should coordinate their parenting styles when raising grandchildren together.

## 1. Introduction

Problem behaviors in children, including both internalizing and externalizing symptoms, have become a critical concern in global public health (Chi & Wang, 2002; Belfer, 2008; J. Liu & Wuerker, 2005). Epidemiological data indicate that the prevalence of these problems ranges from 10.5% to 17.5% (Fu et al., 2019). Early problem behaviors predict various adverse outcomes, such as delinquency during adolescence, violence in adulthood, and the onset of mood and anxiety disorders later in life (Roza et al., 2003; Tremblay et al., 2004). Moreover, children with problem behaviors are more likely to experience peer rejection, face limited employment opportunities in adulthood, and have a higher risk of engaging in criminal behavior (X. Liu et al., 2018; Merhi et al., 2024). Therefore, identifying the factors contributing to the emergence of problem behaviors in children is crucial for promoting their physical and psychological well-being.

According to ecological systems theory, the family serves as the most influential microsystem in an individual’s development and is closely linked to problem behaviors (Szaniecki & Barnes, 2016). In this context, parenting style—a key aspect of the family environment—plays a central role in shaping children’s development. Numerous studies have consistently shown associations between parenting practices and the problem behaviors of children (Aunola & Nurmi, 2005; Hart et al., 2003; Muhtadie et al., 2013). Family systems theory further emphasizes the interdependence of family members, seeing them as subsystems within a larger family unit (Cox & Paley, 2003). As such, parenting behaviors are not isolated but are dynamically shaped by interactions among multiple caregivers. For this reason, understanding the collective influence of both parents on children’s behavior—particularly the impact of parenting consistency—is vital. Consistent, supportive parenting from both parents has been shown to reduce problem behaviors (Lin et al., 2023). In contrast, discordant or unsupportive parenting styles are linked to adverse outcomes, such as anxiety and obesity in children (Sahota et al., 2024).

With increasing life expectancy and more mothers participating in the workforce, grandparental involvement in childrearing has become increasingly common, particularly during early childhood. In Western societies, grandparent caregiving has traditionally been viewed as a response to family crises, such as single parenthood, neglect, or parental unavailability due to substance abuse or work (Ge & Adesman, 2017; Glaser et al., 2013; Jun et al., 2013). Children raised by grandparents often come from high-risk family backgrounds, which complicates understanding their developmental outcomes. In contrast, in Asian contexts, especially in China, grandparent involvement is more widespread (Jun, 2015; Ko & Hank, 2014). Grandparent involvement is common even in intact families where both parents are present (Goh & Kuczynski, 2009). Notably, studies have shown that nearly 58% of Chinese grandparents actively participate in caregiving for their grandchildren (Ko & Hank, 2014). This widespread phenomenon is deeply rooted in cultural traditions that favor multigenerational co-residence. These differences reflect broader cultural values. Chinese culture emphasizes collectivism and relational interdependence, while Western societies tend to prioritize individual autonomy. Thus, further exploration of intergenerational parenting across different cultural contexts is needed.

In certain cultural contexts, such as China, grandparents are deeply involved in childcare responsibilities (Hoang & Kirby, 2020). From a variable-centered perspective, prior research has shown that grandparenting styles significantly affect grandchildren’s developmental outcomes, including survival rates, nutritional status, and emotional and behavioral functioning (Sear et al., 2000; Pettit et al., 2008; Weissman et al., 2016). For example, a study using the Parental Bonding Instrument (PBI) revealed a positive correlation between parental and grandparental bonding. This highlights the intergenerational transmission of caregiving approaches for their grandchildren (Y. Li et al., 2016). Like parental consistency, the combined parenting styles of parents and grandparents form a pluralistic caregiving system. However, we still know little about the latent subgroups of intergenerational (grandparent–parent) parenting profiles and their effects on grandchildren. The grandparent–parent subsystem, a functional unit within the broader family structure, may exert a distinct influence. Inconsistencies or alignments in their caregiving approaches can shape children’s behavioral trajectories. However, there is limited empirical evidence on this topic. Therefore, further research using person-centered analytical methods, such as Latent Profile Analysis (LPA), is needed to uncover underlying patterns in intergenerational parenting and their implications for children’s behavioral outcomes.

In the shared family environment, interpersonal relationships among family members are complex and mutually influential. In the Chinese cultural context, grandparents are not merely auxiliary figures in family life but serve as key executors of daily childcare responsibilities (Ko & Hank, 2014). This cultural tradition grants grandparents a high degree of involvement and authority in family childrearing. However, when grandparents and parents lack alignment in their parenting philosophies, the family may face a “multiple heads of parenting” dilemma, which can lead to intergenerational conflict (X. Li et al., 2020). According to Family Systems Theory (Bowen, 1978), the interaction between grandparents and parents constitutes a critical subsystem within the family, and their cooperation or conflict directly affects the overall stability and functioning of the family system. When the grandparent–parent relationship is harmonious, effective communication can help achieve consistency in parenting logic, thereby enhancing the implementation of caregiving behaviors and providing children with coherent behavioral norms and emotional support—ultimately reducing problem behaviors. Previous research has shown that cooperative caregiving relationships between grandparents and parents can promote grandchildren’s social adaptability and reduce disruptive behaviors (Latham et al., 2018). Furthermore, Emotional Security Theory (Davies & Cummings, 1994) posits that children’s perceptions of the relationship between caregivers within the family system affect their sense of emotional security and coping capacity. When grandparents and parents engage in frequent arguments or mutual invalidation, children may internalize this instability as anxiety, hostility, or confusion about behavioral boundaries, which may, in turn, trigger internalizing or externalizing problem behaviors. Empirical studies have found that family conflict, negative emotional climates, and a lack of cooperative coparenting are associated with increased externalizing behaviors in children (Christopher et al., 2015). In other words, the quality of the grandparent–parent relationship not only reflects the level of family harmony or conflict but also serves as a key mechanism through which intergenerational parenting consistency (or inconsistency) is transmitted to the child. These findings suggest that the quality of the grandparent–parent relationship may be a key mediator between intergenerational parenting styles and children’s problem behaviors.

This study is grounded in the theoretical frameworks of Family Systems Theory (Bowen, 1978), with a focus on exploring the mechanisms through which intergenerational inconsistency in parenting styles affects grandchildren’s development. Based on the theory of intergenerational transmission, we argue that inconsistency in parenting styles disrupts the emotional triangulation within the family system, thereby undermining children’s adaptive functioning (L. Chen et al., 2020). Moreover, although previous studies have highlighted that intergenerational consistency or inconsistency in parenting styles can significantly impact child development, the directionality of inconsistency may lead to distinct developmental mechanisms (Y. Li et al., 2019). For instance, when grandparents exhibit a generally positive parenting style while parents demonstrate harshness or neglect, children may receive conflicting signals from their primary caregivers, resulting in cognitive dissonance and attachment insecurity, which can increase the risk of problem behaviors (Belsky, 2005). Conversely, when parents adopt a supportive and responsive parenting approach but grandparents tend to be controlling or emotionally distant, the grandparents’ negative interactions—as co-caregivers—may still destabilize the family system, induce intergenerational conflict, and indirectly impair children’s emotional and behavioral regulation (Cox & Paley, 1997; He et al., 2023). Therefore, it is of both theoretical and practical significance to investigate how different directions of intergenerational inconsistency influence children’s problem behaviors through the dynamics of family interaction.

## 2. The Current Study

The role of grandparents in Chinese families is increasing. This study aims to investigate intergenerational parenting profiles using a Chinese sample and Latent Profile Analysis (LPA). We have proposed the following hypotheses:

(a) Intergenerational parenting styles can be categorized into distinct latent profiles.
(b) These latent profiles of intergenerational parenting styles have differential impacts on grandchildren’s problem behaviors.
(c) Certain demographic characteristics, such as the number of siblings, the child’s grade level, and the type of grandparent involved, will predict membership in different grandparent–parent parenting profiles.
(d) These parenting profiles are linked to grandchildren’s problem behaviors, with the grandparent–parent relationship mediating this association.

In conclusion, we aim to identify distinct intergenerational parenting patterns and clarify their respective impacts on the behavioral development of children.

## 3. Materials and Methods

### 3.1. Participants and Procedure

After obtaining ethics approval from the first author’s institution, the research team collaborated with school principals in two Chinese provinces to conduct the study. Before completing the questionnaires, all participants were informed of the study’s purpose and procedures. They were assured of their right to provide informed consent and to withdraw at any time. Students completed the questionnaires in school, which were immediately collected upon completion. They were then instructed to take home a study envelope containing materials for their parents. The envelope included a cover letter outlining the study and data usage, informed consent forms for both parents, and separate questionnaires for each parent. Parents were asked to complete the questionnaires independently and return both the completed materials and consent forms to the school the next day.

Prior to data collection, the study was initially designed with the intention of conducting person-centered analyses (e.g., latent profile analysis), which generally require large sample sizes to ensure adequate power and model stability. According to recommendations in the literature, a minimum of 1000 participants is advised for such methods (Nylund et al., 2007; Jung & Wickrama, 2008). Therefore, a total of 1600 questionnaires were distributed, and 1432 valid responses were received, giving a valid response rate of 89.50%. All participants provided written informed consent. The final sample included 648 boys (45.25%), with an average child age of 12.58 years (*SD* = 1.43, range = 10–16 years). The average age of fathers was 39.93 years (*SD* = 5.60) and mothers was 37.97 years (*SD* = 5.51).

### 3.2. Research Tools

#### 3.2.1. Parenting Style

In this study, Gong’s (2005) parenting style questionnaire was used. The same set of items was administered separately to both fathers and mothers. The questionnaire contains 21 items rated on a 5-point Likert scale ranging from 1 (very inconsistent) to 5 (very consistent), covering five dimensions: authoritarian, indulgent, neglectful, affectionate-warm, and trust-encouraging styles. These five dimensions encompass the core characteristics of family upbringing rooted in Chinese cultural traditions, such as “indulgent” and “affectionate-warm,” which align with local parenting practices. Distinct from Western categorizations like “authoritarian” and “permissive,” these dimensions correspond to the study’s focus on “generational differences in parenting approaches.” Developed by Chinese researchers and validated through multiple studies on Chinese parents, the questionnaire’s framework fully incorporates China’s traditional balance between strictness and kindness. This approach better adapts to local cultural contexts compared to Western-introduced tools. In the current study, the Cronbach’s α values for the paternal and maternal versions were 0.85 and 0.79, respectively. Confirmatory factor analysis (CFA) indicated good model fit: for mothers, *χ*^2^/*df* = 4.35, *TLI* = 0.92, *CFI* = 0.98, *RMSEA* = 0.05; for fathers, *χ*^2^/*df* = 4.45, *TLI* = 0.93, *CFI* = 0.99, *RMSEA* = 0.05.

#### 3.2.2. Grandparents’ Parenting Style

The grandparenting style questionnaire, adapted from Gong’s (2005) instrument, was completed by children regarding their closest caregiving grandparent. This 21-item questionnaire uses the same 5-point Likert scale and assesses five dimensions: authoritarian, indulgent, neglectful, affectionate-warm, and trust-encouraging. The core dimensions of grandparents’ parenting and parental parenting share theoretical origins. Therefore, adopting the dimension framework of parental questionnaires ensures comparability in intergenerational parenting approaches, which forms the basis for analyzing differences in these parenting styles. Additionally, children were selected to complete the grandparents’ parenting questionnaire because they serve as direct recipients and observers of grandparents’ and parents’ parenting behaviors, allowing them to perceive daily interactions with grandparents more directly. Cronbach’s α was 0.86. The confirmatory factor analysis (CFA) results demonstrated acceptable model fit: *χ*^2^/*df* = 4.22, *TLI* = 0.93, *CFI* = 0.96, *RMSEA* = 0.05.

#### 3.2.3. Grandchildren’s Problem Behaviors

Children’s problem behaviors were assessed using the Strengths and Difficulties Questionnaire (SDQ; R. Goodman, 1997), consisting of 25 items across five dimensions: emotional symptoms, conduct problems, hyperactivity, peer problems, and prosocial behavior. The Strengths and Difficulties Questionnaire encompasses both internalizing and externalizing problem behaviors, aligning perfectly with the core objective of this study to investigate “the impact of intergenerational parenting on children’s behavioral development.” This instrument comprehensively captures key dimensions of developmental outcomes in children. As an internationally recognized assessment tool, SDQ has been validated for measuring child behavior across diverse cultural contexts (Mu & Du, 2023). Its concise format with 25 items effectively reduces response load for children, making it particularly suitable for the testing scenarios employed in this research. In this study, the prosocial subscale was excluded as the focus was solely on problematic behaviors. Internalizing problems were defined by combining emotional and peer problems, whereas externalizing problems encompassed conduct and hyperactivity issues (A. Goodman et al., 2010). Items were rated on a 3-point scale from 0 (not true) to 2 (certainly true). The internal consistency for the SDQ in this study was acceptable (Cronbach’s α = 0.74), and CFA supported good model fit: *χ*^2^/*df* = 3.09, *TLI* = 0.90, *CFI* = 0.94, *RMSEA* = 0.04.

#### 3.2.4. Grandparent–Parent Relationship

The grandparent–parent relationship was measured using eight questions answered by the students (Yang, 2020), such as “How would you describe your father’s relationship with grandparents?” and “How would you describe your mother’s relationship with grandparents?”. Each item was rated on a 4-point scale ranging from 1 (unfriendly) to 4 (very friendly). In the present study, Cronbach’s alpha for the grandparents–parent relationship was 0.88, indicating high reliability (*χ*^2^/*df* = 2.56, *TLI* = 0.99, *CFI* = 0.99, *RMSEA* = 0.03).

### 3.3. Data Analysis

Descriptive statistics, internal consistency analyses, and multinomial logistic regression were conducted using SPSS 25.0. Latent Profile Analysis (LPA) was performed in Mplus 8.3 to identify distinct profiles of grandparent–parent parenting styles.

To determine the optimal number of latent profiles, models with one to five profiles were evaluated based on several fit indices: the Akaike Information Criterion (AIC), Bayesian Information Criterion (BIC), and sample-size adjusted BIC (adjusted-BIC), with lower values indicating a better model fit (Nylund et al., 2007). Entropy values were also examined, with scores ≥ 0.80 reflecting acceptable classification precision (Jung & Wickrama, 2008). Additionally, the Lo–Mendell–Rubin Adjusted Likelihood Ratio Test (LMR) and the Bootstrap Likelihood Ratio Test (BLRT) were used to compare k-profile models with (k−1)-profile models. A significant *p*-value indicated improved model fit for the k-profile solution (Nylund et al., 2007). To ensure interpretability, profiles with fewer than 5% of the total sample were excluded, as these are considered spurious (Hipp & Bauer, 2006).

After identifying the optimal number of latent profiles, multinomial logistic regression was conducted to examine how these characteristics were associated with different subgroups of children based on intergenerational parenting styles.

## 4. Results

### 4.1. Latent Profile Analysis Results of Intergenerational Parenting Styles

An exploratory Latent Profile Analysis (LPA) was conducted to examine patterns of intergenerational parenting styles among children, based on five observed variables: authoritarian, indulgent, neglectful, affectionate-warm, and trust-encouraging styles. Models specifying one to five latent profiles were evaluated. Table 1 presents the fit indices for each model. Among the five models, the five-profile model yielded the lowest Bayesian Information Criterion (BIC = 268,444.70), suggesting a better statistical fit. However, the Lo–Mendell–Rubin (LMR) test indicated that the four-profile model (LMR *p* = 0.188) fit better than the three-profile model (LMR *p* = 0.242), while the three-profile model fit better than the five-profile model (LMR *p* = 0.584). Entropy values for the three-, four-, and five-profile models were all high (Entropy = 0.96), indicating strong classification accuracy. Upon closer inspection, two of the profiles identified in the four-profile solution were highly similar in structure. When these two profiles were conceptually merged, the resulting profiles were equivalent to those in the three-profile model. Therefore, balancing statistical fit and model parsimony, the three-profile model was retained as the optimal solution for identifying intergenerational parenting style profiles.

To evaluate the accuracy of the latent profile classification, a discriminant analysis was conducted using the five parenting dimensions as predictors. The results indicated that classification accuracy exceeded 93.40% across all grandparent–parent parenting profiles, supporting the reliability of the LPA-derived profiles. To further verify heterogeneity among the profiles, one-way ANOVAs were performed. Significant differences (*p* < 0.001) were found among the three profiles across all five parenting dimensions (see Table 2), confirming the distinctiveness of each profile.

**Figure 1:**
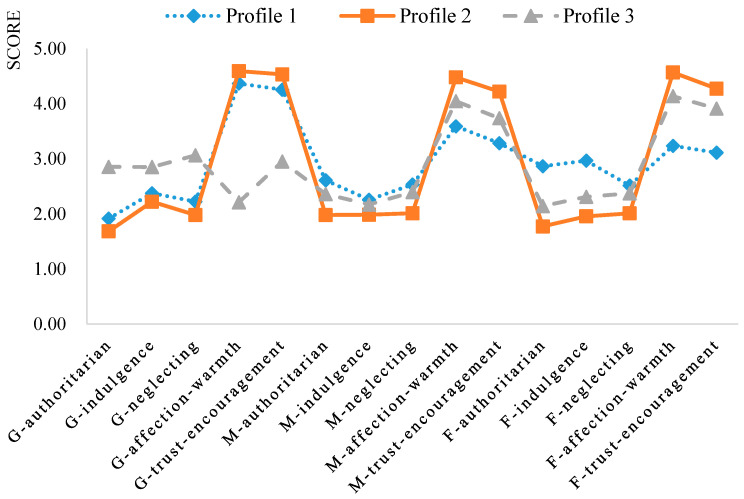
Scores of the three latent profiles on the five dimensions of parenting styles. Note. “G-” = grandparent, “M-” = mother, “F-” = father.

Figure 1 presents the average scores for each latent profile on the five parenting dimensions. In Profile 1, grandparents scored high in affection and trust-encouragement, while parents scored high in authoritarianism, indulgence, and neglect. This profile was labeled “Grandparents Positive–Parents Negative” (GP–PN), representing 18.37% of the sample. In Profile 2, both grandparents and parents scored high in affection and trust-encouragement. This group was labeled “Grandparents Positive–Parents Positive” (GP–PP), accounting for 59.15%. In Profile 3, grandparents scored high on authoritarianism, indulgence, and neglect, whereas parents scored high in affection and trust. This profile was labeled “Grandparents Negative–Parents Positive” (GN–PP), comprising 22.48% of the sample.

### 4.2. Predictors of Latent Profile Membership

Building on the LPA results, we further examined how demographic factors predicted membership in each latent profile, specifically focusing on sibling status, grade level, and grandparent type. A multinomial logistic regression was conducted with the latent profile as the dependent variable, using “GN–PP” as the reference group. Independent variables included only-child status (non-only-child as reference), grade (primary school as reference), and grandparent type (maternal grandfather as reference). As shown in Table 3, significant differences emerged in profile membership based on all three predictors. Children with siblings were more likely to fall into the “GP–PP” profile (*p* < 0.001), suggesting that both grandparents and parents adopt more positive and consistent parenting when children are not only children. Compared to primary school students, junior middle school students were more likely to be in the “GP–PN” and “GP–PP” profiles (*p* < 0.001). Additionally, compared to other grandparents, maternal grandmothers were more frequently associated with the “GP–PN” and “GP–PP” profiles (*p* < 0.05), indicating a greater tendency for warmth, trust, and encouragement in their caregiving (Tanskanen et al., 2023).

### 4.3. The Relationship Between Intergenerational Parenting Styles and the Problem Behaviors of Children

To investigate differences in problem behaviors among children across intergenerational parenting styles, this study analyzed three parenting approaches as independent variables and the problem behaviors as the dependent variable through differential testing (see Table 4). The results revealed significant differences in problem behaviors across parenting styles (*p* < 0.001). Specifically, children in the group with “GN-PP” parenting style exhibited more problem behaviors than those in the group with “GP-PN” parenting style. Children in the group with “GP-PN” parenting style exhibited more problem behaviors than those in the group with “GP-PP” parenting style.

Table 5 presents the bivariate correlations among main study variables. The “GP–PP” profile was negatively associated with child problem behaviors and positively associated with the quality of the grandparent–parent relationship (*p* < 0.001). In contrast, the “GN–PP” profile showed positive correlations with problem behaviors and negative correlations with relationship quality (*p* < 0.001). A significant negative correlation was also found between grandparent–parent relationship quality and children’s problem behaviors (*p* < 0.001).

**Figure 2:**
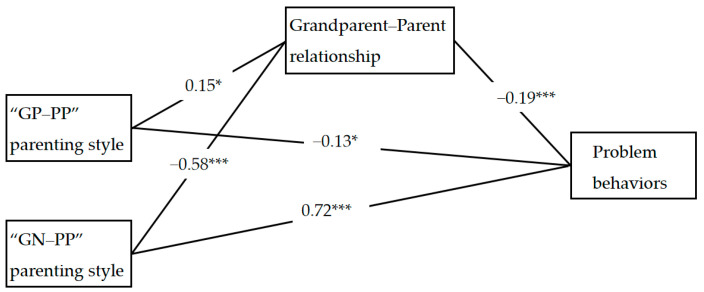
The mediation model. Note. “GP-PP” = “Grandparent Positive–Parent Positive”, “GN-PP” = “Grandparent Negative–Parent Positive”. Note. *** *p* < 0.001, * *p* < 0.05.

We conducted a mediation analysis to examine whether the grandparent–parent relationship mediated the effect of intergenerational parenting styles on children’s problem behaviors. Given the categorical nature of the parenting style variable, dummy coding was used. The “GP–PN” profile served as the reference category (coded as 0). The other two profiles (“GP–PP” and “GN–PP”) were each coded as 1 in separate dummy variables. Figure 2 illustrates the mediation model. The results showed that compared to the “GP–PN” group, the “GP–PP” profile significantly predicted a stronger grandparent–parent relationship (β = 0.15, *p* < 0.05), while the “GN–PP” profile significantly predicted a weaker relationship (β = −0.58, *p* < 0.001). Both the grandparent–parent relationship (β = −0.19, *p* < 0.001) and the “GP–PP” profile (β = −0.13, *p* < 0.05) were negatively associated with child problem behaviors. Conversely, the “GN–PP” profile was positively associated with problem behaviors (β = 0.72, *p* < 0.001). Mediation analysis confirmed that both the “GP–PP” and “GN–PP” profiles had significant indirect effects on child problem behaviors through the grandparent–parent relationship (β = −0.03, *p* < 0.05, 95% CI = [−0.06, −0.003]; β = 0.11, *p* < 0.05, 95% CI = [0.04, 0.12], respectively). Table 6 presents the mediation pathways and their corresponding effect sizes.

## 5. Discussion

### 5.1. The Latent Profile Analysis of Intergenerational Parenting Styles

This study employed Latent Profile Analysis (LPA) to identify three distinct grandparent–parent parenting configurations—GP–PP (Grandparents Positive–Parents Positive), GP–PN (Grandparents Positive–Parents Negative), and GN–PP (Grandparents Negative–Parents Positive)—which represent qualitatively different patterns of family influence. This classification moves beyond the traditional binary of “consistent and inconsistent parenting.”

The GP–PP profile reflects a high-consistency caregiving environment in which both grandparents and parents exhibit warmth and support. The results indicate that grandparents and parents are more likely to adopt similarly positive and congruent parenting styles. This finding aligns with previous research suggesting that caregiving practices tend to converge across caregivers (Y. Li et al., 2019). Parenting behaviors are often transmitted across generations, and grandparents who demonstrate warmth and support may influence parents to adopt similar practices (Safdar & Zahrah, 2016; L. Chen et al., 2020). Such environments foster secure attachment, promote emotional regulation, and reduce the risk of problem behaviors in children.

In contrast, the GP–PN profile involves emotionally supportive grandparents but harsh or negative parenting from parents, which may generate emotional inconsistency and insecurity in children. According to Emotional Security Theory (Davies & Cummings, 1994), exposure to caregivers who convey conflicting emotional signals can lead to cognitive dissonance and relational confusion, undermining children’s emotional regulation and contributing to behavioral disturbances. While grandparents play a meaningful role in the lives of children (Attar-Schwartz et al., 2009), the primary caregiving responsibility typically resides with parents (Leung & Fung, 2014), and their negativity or hostility may override the buffering effects of grandparental support.

The GN–PP (Grandparents Negative–Parents Positive) profile presents a different dynamic. Despite the presence of negative behaviors or outdated practices by grandparents (Gao et al., 2023), the supportive and authoritative parenting style of parents may serve as a psychological buffer, helping children reinterpret or filter the negative influence of grandparents (He et al., 2023). In collectivist cultures such as China, multigenerational co-residence is common, and this compensatory role of parents may be especially crucial for preserving children’s emotional well-being (J. Chen et al., 2025). However, prolonged exposure to grandparental negativity may still lead to chronic stress or intergenerational value conflict, particularly when grandparents hold hierarchical authority in caregiving roles.

These findings highlight that not all intergenerational inconsistencies are equally detrimental. The primary source of influence within the caregiving system—whether grandparents or parents—may determine how discrepancies impact children’s developmental outcomes. Future longitudinal studies are warranted to explore the long-term effects of these interaction patterns.

Multinomial logistic regression analysis revealed that grandparents and parents were more likely to adopt positive parenting styles for non–only children. This may be because parents often place higher expectations on only children, which leads them to adopt more authoritarian approaches. In contrast, parenting styles for non–only children may vary depending on birth order. Compared to firstborns, later-born children often receive more warmth and support from both grandparents and parents (Ling et al., 2023). Additionally, caregivers were more likely to adopt positive parenting styles when children reached junior high school. This developmental period is marked by rapid physical and psychological changes, during which children begin to form a stronger sense of identity and autonomy. In response, parents increasingly emphasize responsibility, social skills, and moral development. As a result, parenting at this stage tends to be more democratic, supportive, and emotionally responsive, with reduced reliance on punishment or overprotection (Chong et al., 2020). Furthermore, maternal grandmothers were more likely to demonstrate affectionate, trusting, and encouraging parenting behaviors. According to the paternity uncertainty hypothesis in evolutionary psychology, grandparents’ investment in their grandchildren is influenced by biological relatedness. Maternal grandmothers, having a confirmed genetic link to both their daughters and grandchildren, typically invest the most. In contrast, paternal grandfathers face the highest level of paternity uncertainty and tend to invest the least (Tanskanen et al., 2023).

### 5.2. The Relationship Between Intergenerational Parenting Styles and the Problem Behaviors of Children

The present study found that children exhibited fewer problem behaviors when both grandparents and parents adopted positive and consistent parenting styles, aligning with previous research (Lin et al., 2023). According to Social Control Theory and Attachment Theory, the family plays a vital role in the socialization of individuals. When grandparents and parents consistently demonstrate care, trust, and responsiveness to children’s needs, strong emotional bonds are likely to form between caregivers and children, reducing the likelihood of behavioral issues (Lee & Park, 2017; Lim, 2018; Teague et al., 2020). In contrast, when parenting styles between generations are inconsistent, children may experience confusion and emotional conflict as they navigate contradictory caregiving approaches. This unstable environment can contribute to the development of problem behaviors. Additionally, research has shown that children’s problem behaviors may, in turn, influence how caregivers interact with and discipline them, highlighting the potential for bidirectional effects (Zhao et al., 2017). These findings underscore the importance of examining the reciprocal and longitudinal dynamics between intergenerational parenting and child behavioral outcomes.

Secondly, this study found that children in the group with “GP-PN” parenting style exhibited more problem behaviors than those in the group with “GP-PP” parenting style. This may stem from the inherent tension between grandparents’ passive parenting and parents’ active parenting. Grandparents ‘negative behaviors directly challenge the “authority” of parents’ positive parenting, creating a state of tension within the family system. To uphold their own parenting philosophies, parents may engage in implicit or explicit conflicts with grandparents. Children, being acutely aware of these intergenerational tensions, become passive recipients of emotional conflicts and express anxiety through problem behaviors. In contrast, grandparents’ positive parenting complements parents ‘passive approach. Their constructive actions don’t challenge parental authority but instead act as a “buffer zone” for negative parenting. For instance, when parents lose their temper over stress, grandparents might comfort the child through companionship. This compensatory support partially offsets the negative impact of parental negativity, thereby reducing overall conflict intensity within the family system.

This highlights the importance of “intergenerational parenting synergy” in family interventions. For households with grandparents involved, it is crucial not only to improve parental parenting approaches but also to reconcile the educational philosophies between grandparents and parents, thereby reducing the “cross-contamination” of negative parenting practices. Particular attention should be paid to the hidden impacts of grandparents’ negative parenting behaviors, which can be mitigated through family communication guidance to reduce risks to children’s development. While this study has identified existing disparities, future research could track family interactions to more precisely capture how intergenerational parenting conflicts influence children’s behaviors in real-time mechanisms.

Furthermore, our mediation analysis revealed that the “GP–PP” profile indirectly reduced problem behaviors through enhanced grandparent–parent relationships, whereas the “GN–PP” profile indirectly exacerbated these problems by weakening intergenerational relationships. When grandparents and parents hold similar views on child-rearing, conflicts are minimized, fostering a more harmonious and supportive caregiving environment. Over time, this consistency contributes to lower levels of behavioral issues in children. Conversely, mismatched parenting styles reflect differences in beliefs about child discipline and caregiving priorities, which can manifest in emotional, behavioral, and academic challenges among children (Derlan et al., 2018). When intergenerational disagreements arise, attention is often diverted from the child’s needs toward conflict resolution and emotional strain. This diversion can lead to psychological fatigue among caregivers, reducing their responsiveness and potentially triggering behavioral issues in children (Christopher et al., 2015; George et al., 2014). In summary, positive and aligned intergenerational parenting enhances grandparent–parent relationships, reduces conflict, and fosters a stable family environment—all of which are critical in mitigating problem behaviors among children.

### 5.3. Practical Implications

Unlike previous studies that primarily focused on grandparents’ individual influence, we innovatively adopted a tripartite intergenerational interaction perspective to reveal for the first time how the alignment patterns of parenting dynamics among grandparents, parents, and children impact child development. This study developed an innovative “Intergenerational Parenting Dynamics Consistency Model,” breaking through the limitations of previous research that only examined single-generation parenting influences.

Building on Family Systems Theory (Bowen, 1978) and developmental situational theory (Lerner, 2002), this study systematically investigates the mechanisms by which latent categories of intergenerational parenting consistency influence children’s problem behaviors. Unlike previous research that predominantly employed variable-centered approaches (e.g., L. Chen et al., 2020), we innovatively applied latent category analysis (LCA) to identify three distinct intergenerational parenting patterns. This discovery not only challenges the unidirectional assumptions of traditional intergenerational transmission theories but also provides a novel analytical framework for understanding complex parenting dynamics within family systems.

The results of this study have clear practical relevance for parenting interventions and policy efforts in China. Inconsistencies between grandparents’ and parents’ parenting styles—especially in the GP–PN and GN–PP profiles—reflect intergenerational gaps in child-rearing values, which are often rooted in historical, cultural, and educational differences (Luo et al., 2020). Such gaps may cause role confusion, undermine family cohesion, and impair children’s emotional security (Xu et al., 2022). To mitigate these risks, co-parenting interventions that include both grandparents and parents are crucial. Programs such as the Triple P–Positive Parenting Program have shown efficacy in improving communication and reducing family conflict and have been successfully adapted in East Asian cultural contexts (Sanders et al., 2014). Additionally, school-based family workshops that address intergenerational expectations and emotional regulation can help harmonize parenting strategies. These interventions are particularly urgent in rural or low-income families, where grandparental caregiving is more common and educational resources are limited (Sun & Yang, 2021). At the policy level, integrating intergenerational parenting modules into community mental health services, parent training policies, and early childhood family guidance programs may strengthen co-parenting alliances, promote child well-being, and reduce problem behaviors related to inconsistent caregiving.

## 6. Limitations and Future Research Directions

This study has several limitations that should be acknowledged. First, the use of cross-sectional data limits the ability to draw causal inferences. Future research employing longitudinal designs would provide a more robust understanding of the influence of intergenerational parenting on problem behaviors among children. Second, the sample was drawn exclusively from primary and secondary school students in two provinces, which may limit the generalizability of the findings. Future studies should recruit participants from more diverse geographic regions to enhance external validity. Another limitation concerns the inconsistency in data sources: grandparenting styles were reported by children, while parenting styles were reported by parents. This discrepancy reflects practical constraints, as grandparents were often unavailable for direct assessment. However, children’s subjective perceptions of caregiving relationships, particularly in multigenerational families, are known to play a critical role in shaping behavioral outcomes (Y. Li et al., 2019; Zhang et al., 2025). Still, the use of different informants may have introduced reporting bias or method variance. Future studies are encouraged to use multi-informant approaches to triangulate the data and enhance measurement robustness.

## 7. Conclusions

In summary, this study yielded three key findings: (1) Intergenerational parenting profiles can be categorized into three types: “grandparent positive-parent negative”, “grandparent positive-parent positive”, and “grandparent negative-parent positive”. (2) When grandparents and parents both adopt positive and consistent parenting styles, children tend to exhibit fewer problem behaviors. In contrast, inconsistency in intergenerational parenting significantly increases the likelihood of such problems. (3) The quality of the grandparent–parent relationship serves as a mediator in the relationship between intergenerational parenting profiles and children’s problem behaviors, highlighting its critical role in family functioning and child development.

## Figures and Tables

**Table 1:** Fit statistics for the Latent Profile Analysis.

Profiles	AIC	BIC	aBIC	Entropy	*p*LMRT	*p*BLRT	Proportions Min (%)
1	283,391.49	284,055.11	283,654.85	-	-	-	
2	275,740.22	276,740.91	276,137.35	0.92	0.751	<0.001	37.53
**3**	**271,138.59**	**272,476.36**	**271,669.49**	**0.96**	**0.242**	**<0.001**	**18.78**
4	268,391.47	270,066.32	269,056.14	0.96	0.188	<0.001	10.38
5	266,432.77	268,444.70	267,231.21	0.96	0.584	<0.001	4.34

Notes. AIC = Akaike information criteria; BIC = Bayesian information criteria; aBIC = adjusted Bayesian information criteria; *p*LMR = *p*-value for Lo-Mendell-Rubin adjusted likelihood ratio test for K vs. K−1 profiles; *p*BLRT = *p*-value for Bootstrapped Likelihood Ratio Test.

**Table 2:** ANOVA for three latent profiles (*M* ± *SD*).

		Profile 1 (*n* = 263)	Profile 2 (*n* = 847)	Profile 3 (*n* = 322)	*F*	*LSD*
Grandparent	Authoritarian	1.91 ± 0.64	1.69 ± 0.53	2.85 ± 0.64	477.86 ***	3 > 1 > 2
	Indulgence	2.38 ± 0.75	2.22 ± 0.71	2.85 ± 0.75	85.60 ***	3 > 1 > 2
	Neglecting	2.23 ± 0.72	1.98 ± 0.62	3.07 ± 0.79	299.75 ***	3 > 1 > 2
	Affection-warmth	4.37 ± 0.63	4.59 ± 0.51	2.95 ± 0.95	747.16 ***	2 > 1 > 3
	Trust-encouragement	4.26 ± 0.61	4.53 ± 0.50	2.95 ± 0.80	823.75 ***	2 > 1 > 3
Mother	Authoritarian	2.61 ± 0.78	1.98 ± 0.55	2.36 ± 0.74	111.52 ***	1 > 3 > 2
	Indulgence	2.26 ± 0.89	1.98 ± 0.67	2.17 ± 0.77	17.64 ***	1 = 3 > 2
	Neglecting	2.54 ± 0.79	2.01 ± 0.61	2.40 ± 0.75	79.31 ***	1 > 3 > 2
	Affection-warmth	3.58 ± 0.88	4.48 ± 0.67	4.04 ± 0.82	158.14 ***	2 > 3 > 1
	Trust-encouragement	3.27 ± 0.96	4.22 ± 0.65	3.72 ± 0.80	177.07 ***	2 > 3 > 1
Father	Authoritarian	2.87 ± 0.50	1.78 ± 0.52	2.14 ± 0.68	389.44 ***	1 > 3 > 2
	Indulgence	2.98 ± 0.79	1.96 ± 0.63	2.31 ± 0.75	221.21 ***	1 > 3 > 2
	Neglecting	2.70 ± 0.66	2.02 ± 0.64	2.37 ± 0.73	114.69 ***	1 > 3 > 2
	Affection-warmth	3.23 ± 0.93	4.57 ± 0.49	4.12 ± 0.87	392.79 ***	2 > 3 > 1
	Trust-encouragement	3.10 ± 0.66	4.27 ± 0.57	3.90 ± 0.76	346.54 ***	2 > 3 > 1

Note. *** *p* < 0.001.

**Table 3:** Multinomial logistic regression analysis for the three latent profiles.

		“GP-PN” Profile		“GP-PP” Profile	
		*OR*	95%*CI*	*OR*	95%*CI*
Sibling number	Non-only-child	1.00		1.00	
	Only-child	0.85	[0.53, 1.63]	0.39 ***	[0.27, 0.57]
Grade	Primary school	1.00		1.00	
	Junior middle school	1.91 ***	[1.25, 2.97]	2.25 ***	[1.87, 3.76]
Grandparent’s type	Maternal grandfather	1.00		1.00	
	Paternal grandmother	1.15	[0.55, 2.43]	1.09	[0.59, 2.05]
	Paternal grandfather	1.29	[0.59, 2.79]	1.38	[0.72, 2.64]
	Maternal grandmother	2.84 **	[1.33, 6.08]	2.20 *	[1.18, 4.11]

Note. “GP-PN” = Grandparents Positive–Parents Negative, “GP-PP” = Grandparents Positive–Parents Positive. *** *p* < 0.001, ** *p* < 0.01, * *p* < 0.05.

**Table 4:** Differences in intergenerational parenting styles in problem behaviors.

		Problem Behaviors (*M* ± *SD*)	*F*	*LSD*
Intergenerational parenting styles	“GP-PN” parenting style (*n* = 263)	11.60 ± 5.14	135.49 ***	“GN-PP” parenting style > “GP-PN” parenting style > “GP-PP” parenting style
	“GP-PP” parenting style (*n* = 847)	10.74 ± 4.83		
	“GN-PP” parenting style (*n* = 322)	16.18 ± 5.62		

Note. “GP-PN” = Grandparents Positive–Parents Negative, “GP-PP” = Grandparents Positive–Parents Positive. *** *p* < 0.001.

**Table 5:** Descriptive statistics and correlations among the main study variables.

	1	2	3	4	5
1. “GP-PN” parenting style	1	—	—	—	—
2. “GP-PP” parenting style	−0.57 ***	1	—	—	—
3. “GN-PP” parenting style	−0.26 ***	−0.65 ***	1	—	—
4. Problem behaviors	−0.05	−0.30 ***	0.40 ***	1	—
5. Grandparent–parent relationship	0.02	0.23 ***	−0.29 ***	−0.29 ***	1
*M*	0.18	0.59	0.22	12.12	27.44
*SD*	0.39	0.49	0.42	5.53	3.87

Note. “GP-PN” = Grandparents Positive–Parents Negative, “GP-PP” = Grandparents Positive–Parents Positive. *** *p* < 0.001.

**Table 6:** Mediation pathways and standardized indirect effects.

Mediation Pathway	Effect	SE	95% *CI*	
			*LLCI*	*ULCI*
“GP–PP” parenting style → Grandparent–parent relationship → Problem behaviors	−0.03	0.01	−0.06	−0.003
“GN–PP” parenting style → Grandparent–parent relationship → Problem behaviors	0.11	0.02	0.04	0.12

## Data Availability

The data that support the findings of this study are available on request from the corresponding author. The data are not publicly available due to privacy or ethical restrictions.

## References

[B1-behavsci-15-01029] Attar-Schwartz S., Tan J. P., Buchanan A. (2009). Adolescents’ perspectives on relationships with grandparents: The contribution of adolescent, grandparent, and parent–grandparent relationship variables. Children and Youth Services Review.

[B2-behavsci-15-01029] Aunola K., Nurmi J. E. (2005). The role of parenting styles in children’s problem behavior. Child Development.

[B3-behavsci-15-01029] Belfer M. L. (2008). Child and adolescent mental disorders: The magnitude of the problem across the globe. Journal of Child Psychology and Psychiatry.

[B4-behavsci-15-01029] Belsky J., Ellis B., Bjorklund D. (2005). Differential susceptibility to rearing influence: An evolutionary hypothesis and some evidence. Origins of the social mind: Evolutionary psychology and child development*.

[B5-behavsci-15-01029] Bowen M. (1978). Family therapy in clinical practice.

[B6-behavsci-15-01029] Chen J., Chen M., Fu Y. (2025). Harsh versus supportive (grand) parenting practices and child behaviour problems in urban chinese families: Does multigenerational coresidence make a difference?. Child & Family Social Work.

[B7-behavsci-15-01029] Chen L., Zhang Y., Lu W. (2020). A review of the influencing factors of parenting styles. Chinese Journal of Clinical Psychology.

[B8-behavsci-15-01029] Chi L., Wang Y. (2002). Theoretical advance on the relations between martal conflict and problem behavors in children. Advances in Psychological Science.

[B9-behavsci-15-01029] Chong L. J., Mirzadegan I. A., Meyer A. (2020). The association between parenting and the error-related negativity across childhood and adolescence. Developmental Cognitive Neuroscience.

[B10-behavsci-15-01029] Christopher C., Umemura T., Mann T., Jacobvitz D., Hazen N. (2015). Marital quality over the transition to parenthood as a predictor of coparenting. Journal of Child and Family Studies.

[B11-behavsci-15-01029] Cox M. J., Paley B. (1997). Families as systems. Annual Review of Psychology.

[B12-behavsci-15-01029] Cox M. J., Paley B. (2003). Understanding families as systems. Current Directions in Psychological Science.

[B13-behavsci-15-01029] Davies P. T., Cummings E. M. (1994). Marital conflict and child adjustment: An emotional security hypothesis. Psychological Bulletin.

[B14-behavsci-15-01029] Derlan C. L., Umana-Taylor A. J., Updegraff K. A., Jahromi L. B. (2018). Mother-grandmother and mother-father coparenting across time among Mexican origin adolescent mothers and their families. Journal of Marriage and Family.

[B15-behavsci-15-01029] Fu C., Niu H., Wang M. (2019). Parental corporal punishment and children’s problem behaviors: The moderating effects of parental inductive reasoning in China. Children and Youth Services Review.

[B16-behavsci-15-01029] Gao F., Bai X. J., Zhang P., Cao H. B. (2023). A meta-analysis of the relationship between parenting styles and suicidal ideation in chinese adolescents. Psychological Development and Education.

[B17-behavsci-15-01029] Ge W., Adesman A. (2017). Grandparents raising grandchildren: A primer for pediatricians. Current Opinion in Pediatrics.

[B18-behavsci-15-01029] George M. W., Fairchild A. J., Mark Cummings E., Davies P. T. (2014). Marital conflict in early childhood and adolescent disordered eating: Emotional insecurity about the marital relationship as an explanatory mechanism. Eating Behaviors.

[B19-behavsci-15-01029] Glaser K., Price D., Di Gessa G., Ribe E., Stuchbury R., Tinker A. (2013). Grandparenting in Europe: Family policy and grandparents’ role in providing childcare.

[B20-behavsci-15-01029] Goh E. C. L., Kuczynski L. (2009). Agency and power of single children in multi-generational families in urban Xiamen, China. Culture.

[B21-behavsci-15-01029] Gong Y. (2005). An exploratory design of the questionnaire of parenting style. Doctoral dissertation.

[B22-behavsci-15-01029] Goodman A., Lamping D. L., Ploubidis G. B. (2010). When to use broader internalising and externalising subscales instead of the hypothesised five subscales on the Strengths and Difficulties Questionnaire (SDQ): Data from British parents, teachers and children. Abnormal Child Psychology.

[B23-behavsci-15-01029] Goodman R. (1997). The strengths and difficulties questionnaire: A research note. Journal of Child Psychology and Psychiatry and Allied Disciplines.

[B24-behavsci-15-01029] Hart C. H., Newell L. D., Olsen S. F., Greene J. O., Burleson B. R. (2003). Parenting skills and social-communicative competence in childhood. Handbook of communication and social interaction skills.

[B25-behavsci-15-01029] He Y., Liu C., Luo R. (2023). Emotional warmth and rejection parenting styles of grandparents/great grandparents and the social–emotional development of grandchildren/great grandchildren. International Journal of Environmental Research and Public Health.

[B26-behavsci-15-01029] Hipp J. R., Bauer D. J. (2006). Local solutions in the estimation of growth mixture models. Psychological Methods.

[B27-behavsci-15-01029] Hoang N. T., Kirby J. N. (2020). A meta-ethnography synthesis of joint care practices between parents and grandparents from Asian cultural backgrounds: Benefits and challenges. Journal of Child and Family Studies.

[B28-behavsci-15-01029] Jun H. J. (2015). Educational differences in the cognitive functioning of grandmothers caring for grandchildren in South Korea. Research on Aging.

[B29-behavsci-15-01029] Jun H. J., Cho K., Park M., Han S., Wassel J. (2013). Longitudinal study of the effect of the transition to a grandparenting role on depression and life-satisfaction. Journal of the Korean Gerontological Society.

[B30-behavsci-15-01029] Jung T., Wickrama A. S. (2008). An introduction to latent class growth analysis and growth mixture modeling. Social and Personality Psychology Compass.

[B31-behavsci-15-01029] Ko P. C., Hank K. (2014). Grandparents caring for grandchildren in China and Korea: Findings from CHARLS and KLoSA. Journal of Gerontology Series B-Psychological Sciences and Social Sciences.

[B32-behavsci-15-01029] Latham R. M., Mark K. M., Oliver B. R. (2018). Coparenting and children’s disruptive behavior: Interacting processes for parenting sense of competence. Journal of Family Psychology.

[B33-behavsci-15-01029] Lee J. Y., Park S. H. (2017). Interplay between attachment to peers and parents in Korean adolescents’ behavior problems. Journal of Child and Family Studies.

[B34-behavsci-15-01029] Lerner R. M. (2002). Concepts and theories of human development.

[B35-behavsci-15-01029] Leung C., Fung B. (2014). Non-custodial grandparent caregiving in Chinese families: Implications for family dynamics. Journal of Children’s Services.

[B36-behavsci-15-01029] Li X., Zhou S., Guo Y. (2020). Bidirectional longitudinal relations between parent-grandparent co-parenting relationships and chinese children’s effortful control during early childhood. Frontiers in Psychology.

[B37-behavsci-15-01029] Li Y., Cui N., Cao F., Liu J. (2016). Children’s bonding with parents and grandparents and its associated factors. Child Indicators Research.

[B38-behavsci-15-01029] Li Y., Cui N., Kok H. T., Deatrick J., Liu J. (2019). The relationship between parenting styles practiced by grandparents and children’s emotional and behavioral problems. Journal of Child and Family Studies.

[B39-behavsci-15-01029] Lim S. A. (2018). The effect of marital conflict between father and mother on their children’s problem behaviors: Mediating effect of children’s attachment security. The Korea Association of Child Care and Education.

[B40-behavsci-15-01029] Lin X., Zhang Y., Liao Y., Xie W. (2023). Socioeconomic status and problem behaviors in young chinese children: A moderated mediation model of parenting styles and only children. Frontiers in Psychology.

[B41-behavsci-15-01029] Ling H., Zhang S. F., Hua S. W., Li H. B., Wang H. H., Yue L. H., Zhang M. L., Zhang J. R. (2023). The influence of grandparents-parents co-parenting relationships on self-supporting behavior of children aged 6~12: The moderating effect of birth order. Chinese Journal of Clinical Psychology.

[B42-behavsci-15-01029] Liu J., Wuerker A. (2005). Biosocial bases of aggressive and violent behavior—Implications for nursing studies. International Journal of Nursing Studies.

[B43-behavsci-15-01029] Liu X., Xing X., Zhang Y. (2018). Executive function and peer relations: The mediating role of externalizing problem behavior. Chinese Journal of Clinical Psychology.

[B44-behavsci-15-01029] Luo Y., Qi M., Huntsinger C. S., Zhang Q. (2020). Grandparent involvement and preschoolers’ social adjustment in Chinese three-generation families: Examining moderating and mediating effects. Children and Youth Services Review.

[B45-behavsci-15-01029] Merhi D., Demou E., Niedzwiedz C. (2024). Mental health and behavioral interventions for children and adolescents with incarcerated parents: A systematic review. Journal of Child and Family Studies.

[B46-behavsci-15-01029] Mu X. X., Du B. F. (2023). Relation of psychological behavioral problems to peer quality and bullying experiences in children from difficult families. Chinese Mental Health Journal.

[B47-behavsci-15-01029] Muhtadie L., Zhou Q., Eisenberg N., Wang Y. (2013). Predicting internalizing problems in Chinese children: The unique and interactive effects of parenting and child temperament. Development and Psychopathology.

[B48-behavsci-15-01029] Nylund K. L., Asparouhov T., Muthén B. O. (2007). Deciding on the number of classes in latent class analysis and growth mixture modeling: A Monte Carlo simulation study. Structural Equation Modeling.

[B49-behavsci-15-01029] Pettit J. W., Olino T. M., Roberts R. E., Seeley J. R., Lewinsohn P. M. (2008). Intergenerational transmission of internalizing problems: Effects of parental and grandparental major depressive disorder on child behavior. Journal of Clinical Child and Adolescent Psychology.

[B50-behavsci-15-01029] Roza S. J., Hofstra M. B., van der Ende J., Verhulst F. C. (2003). Stable prediction of mood and anxiety disorders based on behavioral and emotional problems in childhood: A 14-year follow-up during childhood, adolescence, and young adulthood. American Journal of Psychiatry.

[B51-behavsci-15-01029] Safdar S., Zahrah S. M. (2016). Impact of parenting styles on the intensity of parental and peer attachment: Exploring the gender differences in adolescents. American Journal of Applied Psychology.

[B52-behavsci-15-01029] Sahota N., Shott M. E., Frank G. K. W. (2024). Parental styles are associated with eating disorder symptoms, anxiety, interpersonal difficulties, and nucleus accumbens response. Eating and Weight Disorders—Studies on Anorexia, Bulimia and Obesity.

[B53-behavsci-15-01029] Sanders M. R., Kirby J. N., Tellegen C. L., Day J. J. (2014). The triple p–positive parenting program: A systematic review and meta-analysis. Clinical Psychology Review.

[B54-behavsci-15-01029] Sear R., Mace R., McGregor I. A. (2000). Maternal grandmothers improve nutritional status and survival of children in rural Gambia. Proceedings of the Royal Society B: Biological Science.

[B55-behavsci-15-01029] Sun N., Yang F. (2021). Grandparenting and children’s health-empirical evidence from China. Child Indicators Research.

[B56-behavsci-15-01029] Szaniecki E., Barnes J. (2016). Measurement issues: Measures of infant mental health. Child and Adolescent Mental Health.

[B57-behavsci-15-01029] Tanskanen A. O., Helle S., Danielsbacka M. (2023). Differential grandparental investment when maternal grandmothers are living versus deceased. Biology Letters.

[B58-behavsci-15-01029] Teague S. J., Newman L. K., Tonge B. J., Gray K. M., Team M. (2020). Attachment and child behaviour and emotional problems in autism spectrum disorder with intellectual disability. Journal of Applied Research in Intellectual Disabilities.

[B59-behavsci-15-01029] Tremblay R. E., Nagin D. S., Seguin J. R., Zoccolillo M., Zelazo P. D., Boivin M., Japel C. (2004). Physical aggression during early childhood: Trajectories and predictors. Pediatrics.

[B60-behavsci-15-01029] Weissman M. M., Berry O. O., Warner V., Gameroff M. J., Skipper J., Talati A., Wickramaratne P. (2016). A 30-year study of 3 generations at high risk and low risk for depression. JAMA Psychiatry.

[B61-behavsci-15-01029] Xu X., Xiao B., Zhu L., Li Y. (2022). The influence of parent-grandparent co-parenting on children’s problem behaviors and its potential mechanisms. Early Education and Development.

[B62-behavsci-15-01029] Yang C. (2020). The characteristics of family relationships and its influence on grandchildren’s prosocial behavior under the mode of intergenerational rearing. Doctoral dissertation.

[B63-behavsci-15-01029] Zhang T., Zhou X., Liang L., Yuan K., Bian Y. (2025). Relationship between parent-adolescent discrepancies in perceived parentalwarmth and children’s depressive symptoms and aggressive behavior. Psychological Development and Education.

[B64-behavsci-15-01029] Zhao D., Hou J., Jiang L., Chen Z. (2017). Tracking study of the bidirectional effects of parenting style and externalizing behavior. Journal of Southwest University (Social Sciences Edition).

